# Erectile function and androgen and estrogen beta receptor gene polymorphisms in acromegalic men

**DOI:** 10.1007/s40618-023-02131-2

**Published:** 2023-06-12

**Authors:** F. Pallotti, D. Costa, M. N. Hirsch, V. Mercuri, S. Di Chiano, E. D. Paoli, F. Faja, F. Rizzo, A. Lenzi, D. Paoli, F. Lombardo, P. Gargiulo

**Affiliations:** 1 Laboratory of Seminology‑Sperm Bank “Loredana Gandini”, Department of Experimental Medicine, “Sapienza” University of Rome, Viale del Policlinico 155, 00161 Rome, Italy; 2 Faculty of Medicine and Surgery, University of Enna “Kore”, Contrada Santa Panasia, 94100 Enna, Italy; 3 Department of Experimental Medicine, “Sapienza” University of Rome, Viale del Policlinico 155, 00161 Rome, Italy

**Keywords:** Androgen receptor, Estrogen receptor beta, Acromegaly, Erectile dysfunction, Acromegalic cardiomyopathy

## Abstract

**Purpose:**

Sexual dysfunctions are often experienced by male patients with acromegaly, due to a combination of hypogonadism and other comorbidities, but are a scarcely investigated complication. Erectile dysfunction is also closely related to cardiovascular diseases through endothelial dysfunction. Therefore, this project aimed to assess the prevalence of erectile dysfunction in a population of acromegalic men and evaluate its association with cardio-metabolic disorders, also exploring associations with androgen and estrogen receptor gene polymorphisms.

**Methods:**

Sexually active men aged 18–65 with previous diagnosis of acromegaly were recruited. Clinical and laboratory data were retrospectively collected. Each patient also provided a blood sample for AR and ERβ gene polymorphisms analyses and filled out the IIEF-15 questionnaire.

**Results:**

Twenty men with previous diagnosis of acromegaly (mean age 48.4 ± 10.0 years) were recruited. 13/20 subjects (65%) had erectile dysfunction, but only four had a concurrent biochemical hypogonadism, with no significant correlation with IIEF-15 scores. Total testosterone negatively correlated with sexual intercourse satisfaction domain (*ρ* = − 0.595; *p* = 0.019) and general satisfaction domain (*ρ* = − 0.651; *p* = 0.009). IGF-1 levels negatively correlated with biochemical hypogonadism (*ρ* = − 0.585; *p* = 0.028). The number of CAG and CA repeats in AR and ERβ receptors genes was not significantly associated with IIEF-15 scores or with GH/IGF-1 levels, but a negative correlation between CA repeats and the presence of cardiomyopathy (*ρ* = − 0.846; *p* = 0.002) was present.

**Conclusions:**

Men with acromegaly have a high prevalence of erectile dysfunction, but it does not appear to be correlated with treatments, testosterone levels and AR/ER-beta signaling. Nonetheless, a shorter CA polymorphic trait (ERbeta) is associated with the presence of cardiomyopathy. If confirmed, these data may suggest an association between an incorrect hormonal balance and increased cardiovascular risk in acromegaly subjects.

**Supplementary Information:**

The online version contains supplementary material available at 10.1007/s40618-023-02131-2.

## Introduction

The over-secretion of growth hormone (GH) characterizes acromegaly, a rare chronic disease with a worldwide prevalence of 40–130 per million. Acromegaly is frequently secondary to a pituitary tumor, but clinicians should be aware of its associated comorbidities, such as cardiovascular system disorders, diabetes mellitus (DM), sleep apnea, arthropathy, cancer, and hypogonadism [1]. Early diagnosis and prompt treatment of acromegaly, aimed at obtaining strict control of GH excess, is the best strategy to limit the progression the complications and to prevent premature mortality [2]. Hypogonadism is a relatively frequent comorbidity in patients with pituitary GH-secreting adenoma, but the identification of the critical factors influencing hypogonadism in male acromegaly is currently a shadowy area of research, due to the lack of extensive studies. Nishio et al. have observed that excess GH has a strong correlation with decreased testosterone concentrations in acromegalic males without concomitant hyperprolactinemia. This seems to be due (attributable) to LH suppression, and this condition can be improved after successful surgical treatment [3]. Also as part of the hypogonadism symptoms, male patients with acromegaly may experience erectile dysfunction (ED) and other sexual dysfunctions. These, however, can only be partially justified by hypogonadism in these patients, as other concurrent acromegaly-related factors are also involved in its development (physical disfigurement, psychological comorbidities, vascular endothelial dysfunction, and impaired function of sex hormones and their receptors) [4]. In particular, GH acts as an angiogenic factor and its biological effects are mediated by the action of Insulin-like Growth Factor 1 (IGF-1) that influences endothelial cells with increased nitric oxide (NO) production [5, 6]. NO, through the relaxation of the smooth muscles of the corpora cavernosa, is also the main factor involved in the mechanisms that lead to penile tumescence [7]. This is confirmed by the observation that, in healthy men, GH serum levels appear to increase during tumescence [8]. On the other hand, in acromegalic subjects, it is plausible that the deleterious action on endothelial system of the excess GH/IGF-1 may be associated with alterations in the sexual sphere and erectile function, but this association has been poorly investigated [9]. Furthermore, functional variants of the Androgen Receptor (AR) and Estrogen Receptor (ER) can be associated with altered hormone signaling and, thus, andrological disorders. The AR and ERβ genes, in fact, contain polymorphic fragments with CAG and CA repeats, respectively. The length of these tracts can influence the regulation of receptor activity [10], but the extent of their influence in andrological disorders of acromegalic patients has not been investigated. The multicenter study by Lotti et al. showed a high incidence of ED in acromegalic subjects (24/57), often in association with other cardio-metabolic comorbidities [11]. However, in current clinical practices, ED remains poorly investigated in these patients and, since the association between ED and cardiovascular risk is known, its early identification could improve primary cardio-metabolic prevention and quality of life in these subjects [12]. Therefore, the aim of this project was to:

- Identify the prevalence of ED in a population of acromegalic male subjects and evaluate its association with the presence of any cardio-metabolic disorders;
- Explore any possible associations between the CAG polymorphic trait of the androgen receptor (AR) and the CA polymorphic trait of the estrogen receptor beta (ERbeta) and both ED and cardiovascular disorders.

## Material and methods

### Patients

This study was approved by the “Sapienza” Ethics Committee (Ref. 0945/2020-06-12-2020). We recruited subjects aged 18–65 from the Endocrinology Outpatient Clinics of the Department of Experimental Medicine corresponding to the following criteria:

#### Inclusion criteria

Sexually active men aged 18–65; previous diagnosis of acromegaly, regardless of the clinical course, and/or any surgical or medical treatment.

#### Exclusion criteria

Patients with previous intake of anabolic steroids, antipsychotic/antidepressant drugs, and/or other drugs that are known to interfere with the sexual functioning; subjects suffering from pathologies that could interfere with the evaluation of the study outcomes, such as congenital urogenital malformations, psychiatric pathologies, other untreated endocrinological diseases, Klinefelter syndrome, and/or other known chromosomal or genetic abnormalities, previous antineoplastic treatments.

The diagnosis of acromegaly was performed according to the latest available guidelines, which, in patients with elevated or equivocal serum IGF-1 concentrations, recommend confirmation of the diagnosis by finding lack of suppression of GH to < 0.4 μg/L, following documented hyperglycemia during an OGTT (2 h after 75 g of oral glucose) [12, 13]. Biochemical control of disease was defined by the determination of IGF-1, using an age-related threshold, and GH expressed in ng/mL.

Data deriving from routine clinical and laboratory evaluations has been retrospectively collected. In particular, medical history, general, and andrological examination, anthropometric measurements (height, weight, BMI, waist, and hip circumference), cardiovascular status, biochemical routine, and hormone profile. Each patient provided an additional blood sample for AR and ERβ polymorphisms analyses and filled out the self-administered IIEF-15 questionnaire. Each recruited subject initiated study-related procedures only after acquisition of the informed consent.

### AR/ERbeta polymorphism

A peripheral venous blood sample was collected to extract DNA from leukocytes using the Wizard Genomic DNA Purification Kit (Promega, Madison, WI, USA). After quantification by NanoDrop ND-2000 (Thermo Fisher Scientific, Waltham, MA, USA), the extracted DNA was used to determine the length of the polymorphic fragments in the androgen receptor (AR) and estrogen receptor beta (ERβ) genes (CAG and CA repeats, respectively) by fragment analysis (3500 Genetic Analyzer, Applied Biosystems) as previously described [10]. Supplementary Table 1 shows the primers used for the CA and CAG analyses. Raw data from the capillary electrophoresis were analyzed by Gene Mapper Analysis (Applied Biosystems).

### International index of erectile function (IIEF-15)

Sexual function was evaluated with the self-administered IIEF-15. This multidimensional and validated tool enables the rapid, reliable, and reproducible measurement of several domains of sexual function in clinical trials for ED with high sensitivity and specificity. The questionnaire is composed of 15 items grouped into five domains: erectile function (EF), questions 1–5 and 15; orgasmic function, questions 9–10; sexual desire, questions 11–12; intercourse satisfaction, questions 6–8; and overall satisfaction, questions 13–14. A score below 26 in the EF domain is considered diagnostic for ED [14].

### Statistical analysis

Continuous variables are presented either as mean ± SD or median and 25th–75th percentile, depending on the shape of the distribution curve, which was evaluated using the Kolmogorov–Smirnov test. Categorical variables are presented as absolute numbers and/or percentages. Spearman’s Rank Correlation test was used to investigate correlations among the available variables. A two-tailed *p* value of 0.05 was considered significant. Associations between methylation and the other evaluated parameters were evaluated through linear regressions and generalized linear models. All computations were carried out with the Statistical Package for the Social Sciences (SPSS) 27.0 (SPSS Inc., Chicago, IL, USA).

## Results

### Patients’ demographics

A total of 20 men with previous diagnosis of acromegaly (mean age 48.4 ± 10.0, median 47.0 years) have provided a blood sample for gene polymorphism analysis. Baseline characteristics of the caseload and data on AR/ERβ receptors are shown in Table 1. All subjects at the time of recruitment had GH and IGF-1 levels within normal ranges. All subjects underwent surgery for pituitary GH secreting adenoma (median time from surgery to recruitment was 9 years, ranging between 1 and 32 years). According to the type of treatment, five patients are currently undergoing lanreotide and three pegvisomant treatment; one patient is currently treated with both; five subjects suffered persistent hypogonadotropic hypogonadism after surgery and received androgen replacement treatment; two subjects had concomitant type II diabetes. Finally, eight subjects were diagnosed with obstructive sleep apnea syndrome (OSAS).

**Table 1:** Recruited subjects characteristics at recruitment

	Mean ± SD Median (25th–75th percentile)
Age (years)	48.4 ± 10.0
BMI (kg/m^2^)	25.7 ± 2.8
CAG repeats expression (AR)	22.4 ± 2.5
CA repeats expression (ERβ)	22.4 ± 1.3
Red Blood Cells (× 10^6^/ml)	5.12 ± 0.9
Hemoglobin (g/dl)	14.5 ± 1.5
Hematocrit (%)	44.4 ± 5.3
Glycemia (mg/dl)	91.0 (88–100)
HbA1c (%)	5.6 ± 0.6
Total Cholesterol (mg/dl)	153.8 ± 27.2
HDL (mg/dl)	56.3 ± 11.6
LDL (mg/dl)	80.5 ± 23.1
Triglycerides (mg/dl)	105.0 (79–117)
GH (ng/ml)	1.3 (0.52–1.57)
IGF-1 (ng/ml)	287.4 ± 112.2 (280.0)
FSH (mUI/ml)	4.4 ± 3.3 (4.3)
LH (mUI/ml)	2.4 ± 2.1 (2.6)
Prolactin (ng/dl)	8.0 ± 3.1 (7.6)
Total Testosterone (nmol/l)	13.5 ± 7.1 (10.9)

Data are presented as mean and standard deviations (SD) for normally distributed variables, and median and 25th–75th percentile (in brackets) for non-normally distributed variables

### Sexual functioning

Analysis of the erectile function domain of the IIEF-15 questionnaire showed that 13/20 subjects (65%) reported a degree of ED (EF domain score < 26). Of these subjects, only 4 had concurrent biochemical hypogonadism (total testosterone < 12 nmol/l) but no significant correlation between testosterone values and erectile function scores was detected (Spearman’s *ρ* = − 237; *p* = 0.394). Furthermore, scores from the other IIEF-15 domains indicated that 7/20 patients (35%) had impaired orgasmic function, 14/20 (70%) had impaired sexual desire, while 10/20 (50%) and 7/20 (35%) reported reduced sexual intercourse or general satisfaction, respectively. Sexual intercourse satisfaction, in particular, was significantly and inversely correlated with total testosterone levels (Spearman’s *ρ* = − 0.595; *p* = 0.019); likewise, general satisfaction was negatively correlated with testosterone levels (Spearman’s *ρ* = − 0.651; *p* = 0.009). Regarding disease activity, no significant correlation was detected between IGF-1 and GH levels and IIEF-15 domains. On the other hand, IGF-1 levels negatively correlated with the presence of biochemical hypogonadism (Spearman’s *ρ* = − 0.585; *p* = 0.028). Finally, none of the domains of the IIEF-15 questionnaire appeared associated with OSAS. The scores of the IIEF-15 domains are reported in Table 2.

**Table 2:** IIEF-15 domain scores

IIEF-15 domain	Median (25th–75th percentile)
Erectile function	25.0 (19–28)
Orgasmic function	10.0 (6–10)
sexual desire	8.0 (6–9)
Intercourse satisfaction	11.5 (6–13)
General satisfaction	8.0 (6–8.2)

Data are presented as median and 25th–75th percentile

### Cardiovascular history

No major cardiovascular events were reported in the medical records of the recruited patients, but 16/20 patients received anti-hypertensive treatment and cardiac ultrasound showed the presence of cardiomyopathy in 7/20 subjects (35%). Both the presence of ED and EF domain scores of the IIEF-15 was not significantly correlated with the presence of cardiovascular disease or cardiomyopathy (Spearman’s *ρ* = 0.118 and *ρ* = − 0.191; *p* = 0.653 and *p* = 0.462, respectively). Nonetheless, the presence of cardiomyopathy was significantly negatively correlated with the orgasmic function domain scores (Spearman’s *ρ* = − 0.519; *p* = 0.023), even after adjusting for the age of the subjects (partial correlation *ρ* = − 0.620; *p* = 0.024).

### AR/ERβ polymorphisms

Sequencing of the polymorphic traits of the AR and ERβ receptors showed that the number of CAG and CA repeats in our caseload was comparable to the general population, with a mean of 22.4 ± 2.5 and 22.4 ± 1.3 (22.5), respectively. These genetic polymorphisms were not significantly associated with IIEF-15 scores or with GH/IGF-1 levels. We did find a significant association between CAG repeats and the presence of OSAS (Spearman’s *ρ* = 0.793; *p* = 0.006; age-adjusted 0.756, *p* = 0.018). However, when considering cardiovascular diseases, we found a negative association between CA repeats and the presence of cardiomyopathy (Spearman’s *ρ* = − 0.846; *p* = 0.002; age-adjusted − 0.854, *p* = 0.003). Moreover, we found an interesting positive trend of association between CAG repeats and cardiomyopathy (Spearman’s *ρ* = 0.577; *p* = 0.081).

## Discussion

Acromegaly is capable of interfering with the sexual function of male subjects at multiple levels and in particular through the presence of frequent comorbidities like hypogonadism and vascular/endothelial dysfunctions. Regarding hypogonadism, it is worth to remember that gonadal axis has interesting connections with the somatotropic axis. This intertwined function appears to be characterized by several dynamic redundancies during the transition age, which become evident since puberty, while in adult life, both axes disturbances may impact on reproductive function [15]. In fact, it is known that, both in peri-pubertal boys and in elderly subjects, exogenous testosterone or GnRH administration is associated with an increase in GH levels [16]. Likewise, Leydig and Sertoli cells and primary spermatocytes respond to IGF-1, which may also be secreted under gonadotropin control by the Leydig and Sertoli cells themselves [16]. Furthermore, animal models also show that the lack of IGF-1 compromises maturation and steroidogenic activity of Leydig cells, while exposure to GH may increase responsiveness to gonadotropins through the increase of LH receptors [17, 18]. Excluding the secondary iatrogenic causes (pituitary damage from surgical removal of pituitary GH-secreting adenoma or caused by radiotherapy), hypogonadism may be caused by the mass effect of the adenoma by damaging directly the gonadotropin secreting cell or through the induction of hyperprolactinemia. Moreover, the excess GH/IGF-1 may alter the pulsatile secretion of LH and FSH, further disrupting the gonadal axis [16]. Therefore, due to these strict pathophysiological connections, there is consensus to screen the gonadal function of acromegalic men both at diagnosis and during follow-up [19]. Conversely, despite the obvious link with the hypogonadal state, male sexual function in acromegalic patients is a far more unknown topic. As data on sexual function in men with GH excess is scant, GH- or IGF-1-related effects are difficult to evaluate. It is known that both sexes describe decreased libido and arousal and acromegalic men specifically complain an impaired erectile function. However, the pathophysiological pathway leading to such dysfunctions is difficult to ascertain as it is still unclear whether they are directly related to hormonal excess or secondary to hypogonadism or other comorbidities and treatments. It is also plausible that psychological issues may affect the patients’ sexual function, as acromegaly is often associated with a degree of physical disfigurement and psychological imbalance [20]. Furthermore, data on beneficial effects on the specific surgical or pharmacological approach to acromegaly are far from being fully elucidated, although restoration of normal GH/IGF-1 levels has been associated with improved sexual function [4]. As erectile function is also strictly linked with cardiovascular disease [21], the endothelial function appears an obvious common ground with acromegaly. In fact, on the one hand, physiological levels of IGF-1 can influence endothelial and vascular smooth muscle cells, ultimately reducing vascular tone through nitric oxide production mediated by an increased endothelial nitric oxide synthase (eNOS) expression, which also reduces reactive oxygen species (ROS) levels, limiting oxidative stress [22, 23]. On the other hand, insulin resistance induced by the excess of IGF-1 decreases NO production and thus increases microvascular wall hypertrophy and microvascular dysfunction that correlate with IGF-1 levels [22]. Furthermore, there is evidence indicating that small arteries biopsied from subjects with active acromegaly present a hypertrophic remodeling of the vascular wall as well as a damaged endothelial function, likely due to reduced NO bioavailability [23]. It is thus possible that the common ground of endothelial function could pose as the key risk factor for both cardiovascular disease and impaired erectile function in acromegalic patients. In this light, our paper confirms that patients with acromegaly present a high prevalence of sexual impairment and, specifically, ED. It is also noteworthy that ED does not appear to be associated with specific treatments and testosterone levels, thus shifting the attention toward the vascular etiology, although the small sample size prevents us from acquiring conclusive data. Likewise, erectile function did not appear to correlate with the presence of cardiovascular disease or the presence of acromegalic cardiomyopathy. This is in apparent contrast with another multicenter study [20], but our caseload consisting of subject with biochemically controlled disease has a lower prevalence of hypogonadism and, possibly, milder comorbidities which could weaken the presence of the association. Nonetheless, cardiomyopathy was significantly and negatively correlated with the orgasmic function domain. Although this aspect deserves future investigations, it is possible that the physical signs of acromegaly might impact also on different aspects of sexual functioning, possibly in association of other psychological and physical comorbidities.

An innovative aspect of our study was the investigation of AR or ERβ functional polymorphisms and how variants in the hormone functioning might impact on sexual functioning. First, the sequencing of the AR and ERβ genes polymorphic traits did not show relevant differences in length compared to data available the general Caucasian population. Second, we could determine that these genetic polymorphisms were not significantly associated with IIEF-15 scores or with GH/IGF-1 levels, although this could have been influenced by the small sample size. Nonetheless, we observed interesting significant associations with acromegaly comorbidities, such as the presence of obstructive sleep apnea syndrome. Particularly interesting is that the functional variants of the ER-beta gene were associated with cardiovascular complications: in other words, a shorter CA polymorphic trait is associated with the presence of cardiomyopathy. Although it did not reach statistical significance, we also found a trend of association between a longer CAG trait in the AR and cardiomyopathy. The length of these polymorphic traits has been investigated in various conditions due to their possible role in modulating receptor activity essentially by modifying the transcriptional activity of the AR and ER-beta receptor [10]. CAG repeats in the AR gene, in particular, have been extensively studied and a higher number of CAG repeats has been associated with reduced receptor activity [24]. Fewer data are available for CA trait polymorphism in ERβ gene, but a higher number of CA repeats has been reported to correspond to an increased receptor function [25].

Although this data must be confirmed on a larger sample, it underlines that an incorrect hormonal balance might increase the cardiovascular risk of acromegaly subjects.

We must also highlight the limitations of our study. The retrospective nature of data on comorbidities, as well as the small sample size may limit the generalization of our data. Moreover, the evaluation of the therapy subgroups was not performed to avoid further decrease of statistical power due to the sample size. It must also be noted that enrolled patients had a controlled disease with normal levels of GH/IGF-1, possibly explaining the low presence of hypogonadism. Unfortunately, the absence of values of serum SHBG and free testosterone do not allow to fully evaluate the presence hypogonadism in this caseload.

## Conclusions

This brief report confirms that affected by acromegaly may show a high prevalence of sexual impairment and ED specifically. ED is not correlated with treatments, testosterone levels, and AR/ERbeta signaling, thus shifting the attention toward a vascular etiology, although the small sample size prevents us from acquiring conclusive data. Nonetheless, the exploration of the functional variants of the hormone receptor genes has shown an interesting association with the presence of cardiomyopathy. Since the disease is associated with polymorphic AR and ERbeta variants that are presumed to be linked to weaker hormone signaling, it may suggest that an incorrect hormonal balance might increase the cardiovascular risk of acromegaly subjects.

### Supplementary Information

Below is the link to the electronic supplementary material.

Supplementary file1 Amplification protocol and primers used for Fragment Analysis of AR and ERβ polymorphisms (DOCX 14 KB)

## Data Availability

No data or material to share.

## Code availability

No code to share.
