# Supplementary material for: Erectile function and androgen and estrogen beta receptor gene polymorphisms in acromegalic men
Source: J Endocrinol Invest. 2023 Jun 12;47(1):141–7. doi: 10.1007/s40618-023-02131-2 (PMC10776471; doi:10.1007/s40618-023-02131-2)
Supplement: Supplementary file 1 — Supplementary file1 Amplification protocol and primers used for Fragment Analysis of AR and ERβ polymorphisms (DOCX 14 KB) [file 40618_2023_2131_MOESM1_ESM.docx]

| Gene | Position | Polymorphism | Primers | Conditions of amplification |
| --- | --- | --- | --- | --- |
| ERβ | 14q22-24 | (CA)_n_ intron 5 | 6-FAM-5’-GGTAAACCATGGTCTGTACC-3’  5’–AACAAAATGTTGAATGAGTGGG-3’ | 35 cycles:  95°C 30 sec  61°C 30 sec  72°C 30 sec  72°C 7 min |
| AR | Xq11-12 | (CAG)_n_ exon 1 | 6-FAM-5’-TCCAGAATCTGTTCCAGAGCGTGC-3’  5’-GCTGTGAAGGTTGCTGTTCCTCAT-3’′ | 35 cycles:  94°C 45 sec  59°C 30 sec  72°C 1 min  72°C 7 min |

**Supplementary Table 1** – Amplification protocol and primers used for Fragment Analysis of AR and ERβ polymorphisms.
